# Development of a highly sensitive method based on QuEChERS and GC–MS/MS for the determination of polycyclic aromatic hydrocarbons in infant foods

**DOI:** 10.3389/fnut.2024.1403541

**Published:** 2024-05-10

**Authors:** Mariateresa Ingegno, Rosalia Zianni, Ines Della Rovere, Andrea Chiappinelli, Valeria Nardelli, Francesco Casamassima, Anna Calitri, Maurizio Quinto, Donatella Nardiello, Marco Iammarino

**Affiliations:** ^1^Struttura Complessa di Chimica, Istituto Zooprofilattico Sperimentale della Puglia e della Basilicata, Foggia, Italy; ^2^Department of Agriculture, Food, Natural Resources and Engineering (DAFNE), University of Foggia, Foggia, Italy

**Keywords:** food safety, GC–MS/MS, infant foods, polycyclic aromatic hydrocarbons (PAHs), QuEChERS, validation

## Abstract

Polycyclic aromatic hydrocarbons (PAHs) are environmental contaminants that can be found in various food products, including those intended for infants. Due to their potential health risks, it is crucial to develop sensitive analytical methods for the accurate determination of PAHs in infant foods. This study describes the development and validation of a highly sensitive method for the quantification of European PAH markers, namely benzo[a]pyrene, benzo[a]anthracene, chrysene, and benzo[b]fluoranthene, using gas chromatography–tandem mass spectrometry (GC–MS/MS), in baby food samples. The first step was the optimization of the sample preparation procedure, performed using different methods based on the QuEChERS approach, also testing different extraction solvents. Several factors such as extraction efficiency, selectivity, and recovery were evaluated to choose the most effective procedure for sample preparation. Furthermore, the GC–MS/MS method was optimized, evaluating parameters such as linearity, sensitivity, accuracy, and robustness using spiked infant food samples. The method demonstrated excellent linearities with a correlation coefficient higher than 0.999 over a wide concentration range, and limits of detection and limits of quantification in the range 0.019–0.036 μg/kg and 0.06–0.11 μg/kg, respectively. Extraction recoveries were between 73.1 and 110.7%, with relative standard deviations always lower than 8%. These findings are compliant with the indications of the European Commission (Reg. 836/2011). To assess the applicability of the method to official control activities, a survey was conducted on commercially available infant food products. Four markers were determined in commercial samples belonging to different food categories for infants and young children. The outcome of this monitoring showed that PAH contamination, in all samples, was below the quantification limits. In conclusion, the developed GC–MS/MS method provides a highly sensitive and reliable approach for the determination of PAHs in baby foods. The optimized sample preparation, instrumental parameters, and validation results ensure accurate quantification of 4 PAHs even at trace levels. This method could contribute to the assessment of PAH exposure in infants and it could support regulatory efforts to ensure the safety and quality of infant food products with regular monitoring.

## 1 Introduction

Polycyclic aromatic hydrocarbons (PAHs) are a class of chemical contaminants, comprising over 200 organic compounds, that are originated from the incomplete combustion or pyrolysis of organic matter by natural and anthropogenic processes (1–3). PAHs contain two or more fused benzene rings in linear, angular, or cluster arrangements and they are classified in function of the number of condensed aromatic rings as light (2–3 rings) or heavy (4–6 rings) (4, 5). Due to particular physicochemical properties, such as high lipophilicity, thermostability, low solubility in water, and low biodegradability, these contaminants are ubiquitous and persistent, both in the environment and in the food chain (1, 6). These organic pollutants are highly toxic, mutagenic, carcinogenic, teratogenic, and immunotoxicogenic for several life forms, including humans (7). In particular, the heavy PAHs, such as benzo[g,h,i]perylene, benzo[a]pyrene (BAP), and indeno[1,2,3–c,d]pyrene, because of higher hydrophobicity, are more toxic and stable compared to the light ones (8).

Occurrence and toxicity of PAHs have been evaluated by numerous organizations, e.g., the United States Environmental Protection Agency (U.S. EPA), the International Agency for Research on Cancer (IACR), the Scientific Committee on Food (SCF), the Joint FAO/WHO Expert Committee on Food Additives (JECFA), the International Programme on Chemical Safety (IPCS), and the European Food Safety Authority (EFSA) (9). The two most important groups of PAHs monitored worldwide are the 16 PAHs, listed by U.S. EPA, and the 15 + 1 priority PAHs, defined by SCF for the European Union (EU) (10, 11). The 16 EPA PAHs are the following: acenaphthene, acenaphthylene, anthracene, fluoranthene, fluorene, naphthalene, phenanthrene, pyrene, benzo[a]anthracene, benzo[b]fluoranthene (BBF), benzo[k]fluoranthene, benzo[ghi]perylene, BAP, chrysene (CHY), dibenz[a,h]anthracene, and indeno[1,2,3–cd]pyrene (7, 9). The SCF assessed 33 PAHs and identified 15 PAHs that possess both genotoxic and carcinogenic properties, including 8 high molecular weight PAHs that are also part of the U.S. EPA list. The 15 + 1 EU priority PAHs are benz[a]anthracene (BAA), BBF, benzo[j]fluoranthene, benzo[k]fluoranthene, benzo[ghi]perylene, BAP, CHY, cyclopenta[cd]pyrene, dibenzo[a,h]anthracene, dibenzo[a,e]pyrene, dibenzo[a,h]pyrene, dibenzo[a,i]pyrene, dibenzo[a,l]pyrene, indeno[1,2,3–cd]pyrene, and 5–methylchrysene. These compounds show clear evidence of mutagenicity/genotoxicity and, with the exception of benzo[ghi]perylene, they also show clear carcinogenic effects in various bioassays of experimental animals (9). SFC suggested to use of BAP as a marker of occurrence of carcinogenic PAHs in food because it is based on examinations of PAH profiles in food and on evaluation of carcinogenicity studies (12). PAHs have several negative effects on human health, in particular on reproductive, developmental, cardiorespiratory, and immune systems, also causing asthma or chronic obstructive pulmonary disease and such as breast, skin, bladder, lung, and colon cancer (2). The main routes of human exposure to PAHs are ingestion, dermal, and inhalation pathways, even if food ingestion contributes largely to overall PAH intake (13). Indeed, they can be present in cooked foods as a consequence of the cooking process (12), especially in grilling and deep–frying process, owing to the pyrolysis of fat at higher temperature and adsorption of PAHs emitted from combustion process. In raw foods PAHs may come from the deposition of ambient particles, contaminated soils and water (14). Dietary intake represents a very common and widespread route of exposure specifically for infants and young children, which are particularly vulnerable to food contaminants, due to their different physiological characteristics compared to adults (15–17). Exposure to potentially toxic substances is especially dangerous because of their higher food intake, higher ventilation, and greater body surface area (18). Chronic exposure to PAHs, which unfortunately already begins during gestation through the placenta, continuing with feeding, can lead to delays in cognitive development, disorders of the nervous, urinary and immune systems, and cardiovascular diseases (19). The legislation of the EU Commission established regulation guidelines for the presence of four substances, that is BAP, BAA, BBF, and CHY, known together as PAH4, in food matrices. The lower bound concentration, used as a limit and reference for adherence to safe standards, is calculated as the sum of PAH4 concentrations (20). Regulation (EU) 2023/915 sets maximum levels for PAH4 in foods for infants and young children at 1.0 μg/kg (21).

The extreme heterogeneity of PAHs in foods and their simultaneous presence, combined with their toxicity, require not only a robust regulatory framework but also advanced analytical techniques. The distribution of PAHs is influenced by different physical states of foods, therefore extraction and analysis methods must be adapted to the specific matrices to detect and quantify these contaminants (3). To provide repeatable data and satisfy legal criteria, proper sample preparation with adequate extraction procedures and improved cleaning strategies are required. Soxhlet extraction is probably the most widely used traditional extraction technique for PAHs from a wide range of foodstuffs. However, this conventional approach is laborious, time–consuming and requires great amounts of organic solvents (22, 23). To date, automated extraction techniques for PAHs in food have achieved popularity through their increased efficiency, shorter analytical times, and environmentally friendly characteristics. They include accelerated solvent extraction (ASE), microwave–assisted extraction (MAE), supercritical fluid extraction (SFE), high-temperature distillation (HTD), and fluidized–bed extraction (FBE) (1). Among the clean–up procedures, column chromatography, such as gel permeation chromatography (GPC), Florisil, and silica gel, is the standard approach which is characterized by an extensive quantity of reagents, solvents, and materials. The solid–phase extraction (SPE), along with dispersive liquid–liquid microextraction (DLLME), solid–phase microextraction (SPME), magnetic solid–phase extraction (MSPE), and QuEChERS (Quick, Easy, Cheap, Effective, Rugged, and Safe) are the clean–up procedures with high enrichment factor, automation capability, and less exposure to organic solvents (9). In particular, the QuEChERS method is based on the green chemistry principles of multi-residue analysis, with several advantages such as lower costs, fewer solvents, time savings, increased yield, and great extraction performance (24–26). The QuEChERS strategy is characterized by great versatility and, for this reason, it has been systematically employed in routine analytical determinations of a wide range of analytes (e.g., pesticides, mycotoxins, pharmaceutical residues, illicit drugs, etc.) in environmental, food, feed, pharmaceutical, biological, and forensic matrices (27). The experimental layout of the QuEChERS method includes two steps: a solid–liquid extraction/partitioning with a salting–out effect and a dispersive solid-phase extraction (d–SPE) for sample clean–up. The extraction is performed with acetonitrile partitioned from an aqueous matrix using MgSO_4_ and NaCl, followed by dispersive-SPE (d-SPE) with MgSO_4_, a primary secondary amine (PSA), and another sorbent such as octadecyl silica (C18) and graphitized carbon black (GCB) (24, 28).

In the QuEChERS d-SPE method, the choice of adsorbents is critical for the reduction of matrix interference in the following chromatographic analysis. In the original method proposed by Anastassiades et al. (24), the first extraction step was carried out under unbuffered conditions with acetonitrile as solvent, but this approach had some limitations. To overcome these drawbacks, citrate buffer (with a relatively low buffering capacity) (29) and/or acetate buffer (with a strong buffering capacity) (30) were used to enhance the extraction efficiency. Another important factor is the nature of the extraction solvent, which can be modified according to the target analyte. Acetonitrile is an excellent separator from water, after the salt addition, therefore it is suitable to extract the broadest range of organic compounds, without co-extraction of large amounts of lipophilic material, in different matrices (3, 27). However, to improve the extraction efficiency of target analytes some solvent modifications have been proposed, namely acidification or combination of acetonitrile with isooctane and/or ethyl acetate (31–33).

PSA is typically used as sorbent of the d-SPE step to remove fatty acids, sugars, organic acids, lipids, and some pigments. C18 is particularly effective for the removal of high lipid contents while GCB is used to remove co-extracted pigments, namely carotenoids and chlorophyll, typical from highly pigmented matrices (27, 33). For other complex matrices, more oriented adsorbents based on new materials have been developed, such as zirconium-coated silica (32). Furthermore, modifications of the QuEChERS procedure in terms of solvents, salts, and sorbents, are continuously proposed to improve and broaden even more the range of applications from food samples (9).

In this work, different extraction and purification methods, principally based on molecularly imprinted polymers (MIP) and QuEChERS with Enhanced Matrix Removal-Lipid (EMR-lipid), were considered. MIPs are a class of highly cross-linked polymer-based molecular recognition elements engineered to bind one specific target compound or a class of structurally related compounds with high selectivity. The MIP material is designed with cavities that are sterically and chemically complementary to the target analytes. As a result, multiple interactions (e.g., hydrogen bonding, ionic, Van der Waals, hydrophobic) can take place between the MIP cavity and the analytes. On the other side, EMR-Lipid is a new product used as a d-SPE to remove lipids. This technology, based on size exclusion and hydrophobic interactions, is very promising for the selective removal of lipids, ensuring minimal loss and ion suppression of target analytes, and improving method reliability and ruggedness. Moreover, modified-QuEChERS procedures were tested, also using EMR-Lipid in combination with different extraction and purification salts.

For the identification and quantification of PAHs in a wide range of food matrices, common analytical approaches are adopted, such as high–performance liquid chromatography (HPLC) with an ultraviolet (UV) array detector photodiode array (PDA), and gas chromatography (GC) coupled to a flame ionization detector (FID). However, these techniques are not sensitive, not selective, time-consuming, and labor-intensive (9). High-resolution mass spectrometry (HRMS) detectors allow better performance than DAD and FLD, and for this reason, they are widely used in PAH determination in food matrices (1). In particular, GC coupled to tandem mass spectrometry (MS/MS) is the most common analytical tool, mainly due to its sensitivity, accuracy, and convenience even in official methods (34, 35). Current literature provides several investigations focused on the extraction and analysis of PAHs in baby food, comprising both traditional and novel approaches (32). Moazzen et al. (17) proposed the good performances of MSPE method coupled to GC–MS/MS to detect PAHs in the Irian market. Recently, Prata et al. (36) provided a QuEChERS-based extraction procedure based on different salts composition in addition to MgSO_4_ and PSA, such as Florisil, C18 and zirconium-coated silica. All these approaches are adequate for the determination of PAHs in baby food, but they are quite laborious also requiring particular laboratory equipment for sample preparation, which is not always available.

In this study, the preparative procedure based on modified-QuEChERS for the extraction and purification of four regulated PAHs was optimized, and the following efficient GC–MS/MS analytical method was validated to detect and quantify the target analytes in baby foods. Compared to the current methods, the proposed procedure involves a double initial extraction with acetonitrile and extract freezing at −20°C, before clean-up. Therefore, a method based on a simple modification of standard QuEChERS protocol for contaminants analysis was optimized and used for the first time to analyse the main PAHs in several types of baby food. The developed method was found to be accurate, efficient, sensitive, and selective for the analysis of food products of different types, based on meat, fish, legumes, and vegetables, subject to official control for the presence of BAP, BAA, BBF, and CHF. To the best of our knowledge, there is a lack of information regarding the determination of PAHs in the Italian markets. Therefore, this procedure was used to analyze food samples present on the Italian market and intended for infant nutrition, demonstrating its suitability for baby food control. Cooking methods, i.e., microwave and steam cooking, were also considered to verify PAH contamination as a consequence of heat treatments.

## 2 Materials and methods

### 2.1 Samples and chemical standards

A total of 60 samples, consisting of meat (chicken) and mixed meat, mixed fish, and mixed legumes were used for validation. A total of 100 samples, consisting of meat (chicken, beef, lamb, ham) and mixed meat, fish (salmon and trout) and mixed fish, legumes (white beans, chickpeas, lentils) and mixed legumes, and vegetables (green peas and courgettes) were analyzed for monitoring purposes. All samples, listed in Table 1, were purchased in local markets and subsequently stored in their original packaging, properly labeled, and refrigerated (4 ± 1°C) until analysis. In Italy, in accordance with the European Regulation (37), the dietary guidelines suggest introducing homogenized products into infant complementary feeding, particularly during weaning, thanks to their consistency and specific composition.

**Table 1:** Declared composition of investigated baby food matrices.

Matrix	Protein content (%)	Salt content (%)	Carbohydrates content (%)	Total lipid (%)	Fatty saturated (%)
Chicken	6.1	0.08	6.3	2.4	1.6
Beef	5.8	0.06	5.7	3.7	1.3
Lamb	5.5	0.05	6.8	3.1	1.5
Ham	7.9	0.20	8.7	3.6	1.2
Mixed Meat	7.5	0.10	6.6	2.1	0.5
Salmon	4.0	0.03	8.7	2.4	0.5
Trout	3.7	0.03	9.2	1.4	0.3
Mixed Fish	4.0	0.08	10	2.4	0.5
White beans	3.5	0.01	6.4	0.005	0.001
Chickpeas	2.5	0.01	4.9	0.9	0.1
Lentils	2.4	0.04	6.5	0.2	0.001
Mixed legumes	3.4	0.05	5.9	0.004	0.002
Green peas and courgettes	1.9	0.003	7.9	0.3	0.002

Analytical grade cyclohexane, ethyl acetate, and acetonitrile were supplied by Merck Life Science s.r.l. (Darmstadt, Germany). Isooctane was purchased from Panreac Química S.L.U. (Castellar del Vallès, Barcelona, Spain). Deionized water (18.2 MΩ/cm) was obtained from a Milli-Q purification system (Millipore, Milan, Italy). Polychlorinated biphenyl (PCB) 209 (purity >99.0%; 10 mg/L in isooctane, Lab Instruments Castellana Grotte, Bari, Italy) was used as internal standard. From this stock standard solution, PCB 209 working standard solutions of 1,000 μg/L in isooctane were prepared and used to spike food samples at 100 μg/L. Within the subsequent monitoring survey of PAH levels in tested samples, PCB 209 was added to the samples before extraction, to perform reliable quantifications of target analytes with correction of errors due to ion suppression/enhancement caused by the presence of matrix co-extracts in the injector and ion source. Certified mix standard solution, indicated as IL8 constituted by benzo[a]pyrene (BAP), benz[a]anthracene (BAA), benzo[b]fluoranthene (BBF), chrysene (CHR) and their relative deuterated (PAHs-d12), that is BAP-d12, BAA-d12, BBF-d12 and CHR-d12 (purity >99.0%; 100 mg/L in toluene, Lab Instruments Castellana Grotte, Bari, Italy) was used for the method validation. Working standard solutions of 1,000 μg/L, 100 μg/L, 25 μg/L, and 10 μg/L were obtained by diluting the stock IL8 in isooctane. All standard solutions were stored at −20°C and were taken out to allow them to reach room temperature and sonicated before use. Matrix-matched calibration (MMC) curves were obtained using 0.1, 0.5, 1.0, 2.0, 5.0 μg/L working standard solutions of IL8 in the blank sample. The presence of the matrix effect was considered in the optimization and validation steps and in the monitoring study. MMCs were used because the different substances investigated had a different composition from each other, i.e., lipid, protein, carbohydrate and salt content (Table 1).

SupelMIP SPE – PAHs cartridges (50 mg/3 mL) were purchased from Merck Life Science s.r.l. (Darmstadt, Germany). Bond Elut Enhanced Matrix Removal - Lipid dispersive SPE (Bond Elut-EMR lipid dSPE) Agilent Technologies were purchased from Analitica sas (Gioia del Colle, Bari, Italia).

For QuEChERS procedures, all extraction and purification salts were listed below and were supplied by Lab Instruments (Castellana Grotte, Bari, Italy):

- QuE-Lab® EMR dSPE Lipid Tube (15 mL) with the following composition: 0.40 g NaCl and 1.60 g MgSO_4_;
- QuE-Lab® Citrate tube (15 mL) with the following composition: 4.00 g MgSO_4_, 1.00 g NaCl, 0.50 g Na_2_HC_6_H_5_O_7_·1.5H_2_O and 1.00 g C_6_H_5_Na_3_O_7_·2H_2_O;
- QuE-Lab® PSA Tube (15 mL) with the following composition: 950 mg MgSO_4_ and 150 mg Primary secondary amine sorbent (PSA);
- QuE-Lab® PSA/C18 Tube (15 mL) with the following composition: 900 mg MgSO_4_, 150 mg PSA, and 150 mg C18 end-capped (C18EC);
- QuE-Lab® LLE tube EMR method (50 mL), with the following composition: 3.00 g NaCl, 0.50 g Na_2_HC_6_H_5_O_7_·1.5H_2_O and1.00 g C_6_H_5_Na_3_O_7_·2H_2_O;
- QuE-Lab® dSPE MgSO_4_ tube EMR method (15 mL), with the following composition: 0.50 g MgSO_4_.

### 2.2 Extractions and clean-up procedures

The optimization of PAH extractions and clean-up protocols were performed on smoked salmon samples and chicken baby foods, following what was suggested by literature and on the base of our earlier knowledge relevant to the multi-residual analysis of foodstuffs (38, 39). During the development of analytical procedures, an ultrasonic bath (LIARRE s.r.l., Casalfiumanese, Bologna, Italy), a TX4 Digital vortex mixer (Velp Scientifica, Usmate Velate, Italy) and a BKC-DL5M centrifuge (BiobaseMeihua Trading Co., Ltd., Jinan, China) were used to sonicate, vortex and centrifuge, respectively.

#### 2.2.1 Protocol I

SupelMIP SPE – PAH for extraction and clean-up. Aliquots of 2.5 g of homogenized samples were weighed into 10 mL screw-cap vials, 7.5 mL of cyclohexane were added, and then stirred for 2 min with a TX4 Digital VortexMixer (Velp Scientifica, Usmate, Italy) to disperse the sample. Afterwards, the mixture was sonicated for 20 min using an ultrasonic bath (LIARRE s.r.l., Casalfiumanese, Bologna, Italy), and centrifuged at 1,.500 rpm for 15 min at 4°C with a BKC-DL5M centrifuge (BiobaseMeihua Trading Co., Ltd., Jinan, China). A SupelMIP column was conditioned with 1 mL of cyclohexane, then the sample was loaded, discarding the first eluate, avoiding its complete drying. After column washing with 2 mL of cyclohexane, for the elution of PAHs, 3 mL of ethyl acetate were used, collecting the eluate in a 10 mL glass vial, completely drying the column under vacuum. The extract was evaporated to dryness at 55°C under N_2_ flow using an automated solvent evaporation system TurboVap® II (Biotage AB, Uppsala, Sweden). Finally, the dried extracts were suspended with 0.5 mL of working standard solution of PCB 209 and transferred into a glass vial for the following GC–MS/MS analysis in duplicate.

#### 2.2.2 Protocol II

QuEChERS method using Bond Elut-EMR lipid dSPE for extraction and purification salts a) for clean-up. Aliquots of 5.0 g of homogenized samples were weighed into a 10 mL polypropylene tube, and 10 mL of acetonitrile were added. The mixture was vortexed for 2 min and centrifuged at 3,000 rpm for 5 min at 4°C. The resulting supernatant was then added to a tube containing 1.0 g of EMR-Lipid adsorbent and was vortexed for 2 min. The phases were separated by centrifugation at 3,000 rpm for 5 min at 4°C, and subsequently, the supernatant was put into a dispersive SPE tube containing a mixture of salts MgSO_4_ and NaCl, vortexed for 2 min and then centrifuged at 3,000 rpm for 5 min at 4°C. The supernatant (8 mL) was evaporated to dryness under a N_2_ stream at 55°C.

#### 2.2.3 Protocol III

QuEChERS method using Bond Elut-EMR lipid dSPE for a double extraction and purification salts a) for clean-up. This protocol is the same as *Protocol II*, but the extraction procedure was repeated twice. Indeed, this step was carried out by adding acetonitrile and Bond Elut-EMR lipid two times consecutively, therefore the final volume of supernatant to be evaporated to dryness was 16 mL.

#### 2.2.4 Protocol IV

QuEChERS method using dispersive salts b) for extraction step, and purification salts c) for clean-up. In this procedure, a double extraction with acetonitrile was performed and then the resulting supernatant was extracted with the following dispersive salts MgSO_4_, NaCl, Na_2_HC_6_H_5_O_7_·1.5H_2_O and C_6_H_5_Na_3_O_7_·2H_2_O. Afterward, the mixture was vortexed for 2 min and the phases were separated by centrifugation 3,000 rpm for 5 min at 4°C;, subsequently, the supernatant was purified with a mixture of salts MgSO_4_ and PSA, vortexed for 2 min, and then centrifuged at 3,000 rpm for 5 min at 4°C. The supernatant (16 mL) was evaporated to dryness under an N_2_ stream at 55°C.

#### 2.2.5 Protocol V

QuEChERS method using dispersive salts b) for extraction step and purification salts d) for clean-up. This protocol is the same as Protocol IV for the three extractions, but the clean-up was performed using MgSO_4_, PSA, and C18EC as dispersive salts. The supernatant (16 mL) was evaporated to dryness under an N_2_ stream at 55°C.

#### 2.2.6 Protocol VI

QuEChERS method using dispersive salts e) for extraction step and Bond Elut-EMR lipid dSPE and purification salts f) for clean-up. In this procedure, a double extraction with acetonitrile was performed and then the resulting supernatant was extracted with the following dispersive salts NaCl; Na_2_HC_6_H_5_O_7_·1.5H_2_O and C_6_H_5_Na_3_O_7_·2H_2_O. The extracted supernatant, firstly with Bond Elut-EMR lipid and after with MgSO_4_, was then purified. Between the two purification steps, the sample was vortexed for 2 min and centrifuged at 3000 rpm for 5 min at 4°C. The supernatant (5 mL) was evaporated to dryness under an N_2_ stream at 55°C.

#### 2.2.7 Protocol VII

QuEChERS method using Bond Elut-EMR lipid dSPE and purification salts a) for clean-up. In this procedure, a double extraction with acetonitrile was performed and then the resulting supernatant was again extracted with EMR-lipid. Afterward, the supernatant was refrigerated at −20°C for 1 h and then purified with MgSO_4_ and NaCl, vortexed for 1 min, and then centrifuged at 3,000 rpm for 10 min at 4°C. The supernatant (16 mL) was evaporated to dryness under an N_2_ stream at 55°C.

Finally, all the dried extracts obtained from protocols II up to VII were suspended with 1.0 mL of working standard solution of PCB 209, as internal standard, and transferred into a glass vial for the following GC/MS/MS analysis in duplicate.

### 2.3 Gas chromatography/tandem mass spectrometry analyses (GC–MS/MS)

An Agilent 7693A Automatic Liquid Sampler and an Agilent 8,890 N gas chromatograph coupled with an Agilent 7,000 Triple Quadrupole detector (Little Falls, DE, United States) were used for GC–MS/MS analyses. Data were acquired using MSD ChemStation software (Agilent, Little Falls, DE, United States). The GC–MS/MS operating conditions are shown in Table 2. Backflushing was used to prevent contamination with compounds strongly retained in the primary column by reversing a continuous flow of carrier gas, thus avoiding, through the second column, reaching the MS detector. According to European directive (40), the number of identification points that GC–MS/MS can earn, namely three points, one for ion precursor and 1.5 for each of two daughter ions. The identification point can be five if there are two precursor ions, each with 1 daughter of five points. The list of precursor ions, both quantifiers and qualifiers, their transitions, as well as the chosen collision energies (CEs), were shown for each of the 4 PAHs and the 4 PAHs-d12, in Table 3.

**Table 2:** Operating conditions of GC–MS/MS.

Chromatographic parameters	
Liner	Ultra Inert Liner of 78.5 mm, ID 4 mm, OD 6.47 mm, volume 900 μL and single taper (Agilent technologies Little Falls, DE, United States)
Column 1 and Column 2	Ultra Inert 15 m x 0.25 mm, 0.25 μm film thickness (Agilent J&W. Scientific, Folsom, United States)
Carrier gas and flow mode	Helium carrier gas (99.999%); Flow in Colum 1: 1.0 mL/min Flow in Colum 2: 1.2 mL/min
Injection mode, temperature, and volume	Splitless mode, 280°C, 1 μL, hold time 3.0 min
Oven temperature	60°C (held 1.0 min); 170°C (rate 40°C/min, held 3.75 min) and 310°C (rate 10°C/min)
Blackflush	320°C (held 5.0 min)
Run time	18 min + 5 min blackflush

Mass spectrometer parameters	
Detector	MS operating in electron ionization mode: 70 eV
	Temperature of MS1 and MS2: 150°C
	Source temperature: 280°C
	Transfer line: 290°C
	Acquisition in Multiple Reaction Monitoring mode (MRM),
	Resolution MS_1_ = 1.5
	Resolution MS_2_ = 1.5

**Table 3:** List of quantifier and qualifier ions (m/z) used in GC–MS/MS method for PAHs, PAHs-d12, and PCB-209 determination.

Analyte	RT^a^	Quantifier ion	Transition	CE^b^	Qualifier ion	Transition	CE^b^
BAP	16.97	252	252 → 250	45	252	252 → 224	60
BAP-d12	16.92	264	264.1 → 260.1	40	265.1	265.1 → 261.1	40
BAA	13.93	228	228 → 226	40	228	228 → 227	25
BAA-d12	13.88	240	240 → 236	40	240	240 → 238	30
BBF	16.33	252	252 → 248	60	252	252 → 224	60
BBF-d12	16.28	264.1	264.1 → 260.1	40	265.1	265.1 → 261.1	40
CHR	14.02	228	228 → 226	40	228	228 → 227	25
CHR-d12	13.96	240	240 → 238	20	240	240 → 236	38
PCB 209	17.08	497.7	497.7 → 427.7	30	495.8	495.8 → 425.7	30

a RT Retention time (min).

b CE collision energy (V).

## 3 Results and discussion

### 3.1 Optimization of extraction and clean-up procedure

The identification of BAP, BAA, BBF, and CHR in GC–MS/MS analysis was performed by comparing the retention time of each compound with that of the relevant standard and verifying the presence of precursor ions (Table 3). The ratios between the peak areas of quantifier ions and PCB 209 were used for the compound quantification.

**Figure 1:**
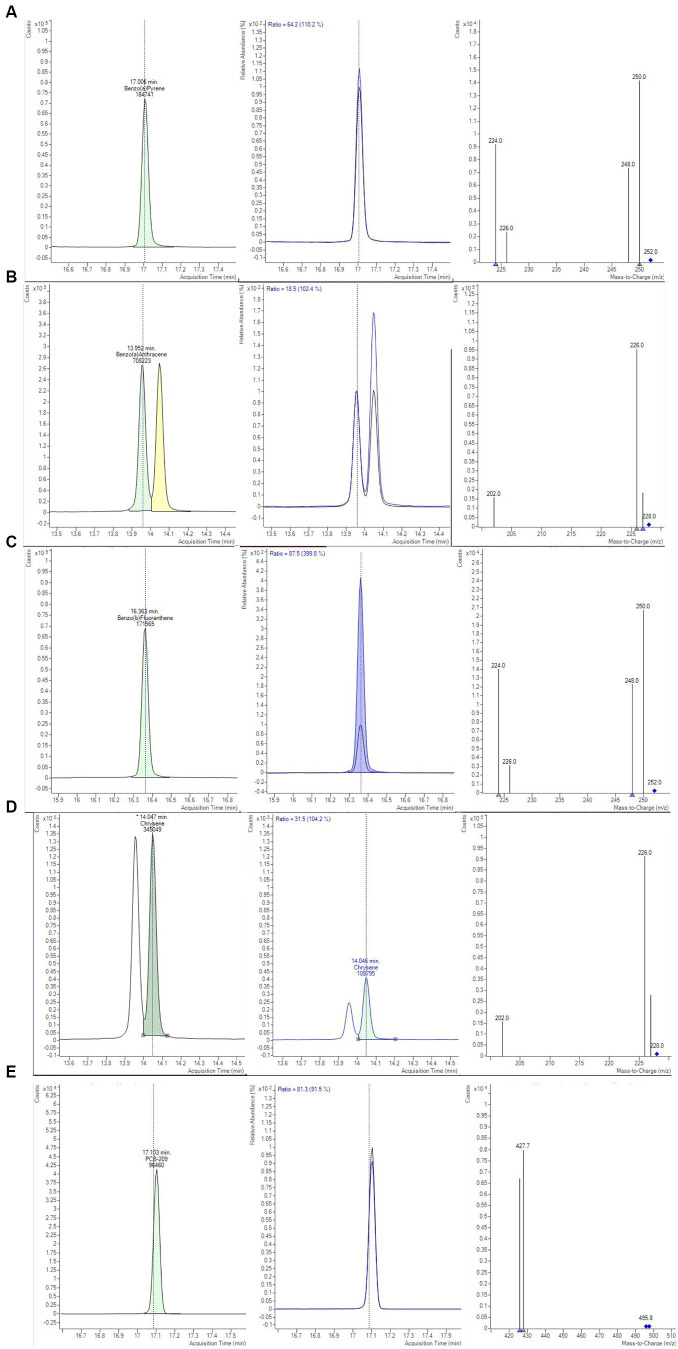
Chromatograms and mass spectra of quantifier and qualifier ions (m/z) used in GC-MS/MS method for **(A)** BAP, **(B)** BAA, **(C)** BBF, **(D)** CHR, **(E)** PCB-209 determination, obtained from smoked salmon samples, extracted with Protocol II based on QuEChERS method (Bond Elut-EMR lipid dSPE for extraction and purification salts for clean-up, see text for details).

The first two extraction and clean-up protocols were tested using MMC curves with smoked salmon samples. MMC curves of SupelMIP SPE and QuEChERS based on Bond Elut-EMR lipid dSPE showed correlation coefficients ≥0.99. The recovery percentage at 2 μg/kg was also evaluated, carrying out 3 replicates for both protocols, also comparing two different solvents, i.e., acetonitrile and toluene, for the preparation of the IL8 working standard solution used to spike the samples. The results, shown in Table 4, demonstrated that the QuEChERS approach gave higher recovery values (71.4–83.0%) compared to SupelMIP SPE (29.8–48.8%). In Figure 1, the chromatographic separation and mass spectrometric identification of the analytes were reported for protocol based on the QuEChERS procedure. Therefore, the QuEChERS method was selected as the best procedure and was further optimized, modifying the extraction and purification steps in the other 5 Protocols (III, IV, V, IV, and VII). No significant differences emerged changing the solvent for standard solutions preparation, therefore, considering the high toxicity of toluene compared to acetonitrile, the latter was selected for further experiments.

**Table 4:** Mean values (*N* = 3) of recovery % of smoked salmon samples, spiked at 2 μg/kg, obtained from Protocol I and II, using IL8 working standard solution prepared with two solvents, toluene and acetonitrile, respectively.

Extraction and clean-up procedure	Solvents	BAA	BAP	BBF	CHR
*Protocol I*	Toluene	40.3%	29.6%	33.4%	40.4%
	Acetonitrile	48.8%	36.5%	40.4%	48.1%
*Protocol II*	Toluene	80.6%	71.4%	77.6%	83.0%
	Acetonitrile	80.3%	71.4%	78.2%	80.0%

**Figure 2:**
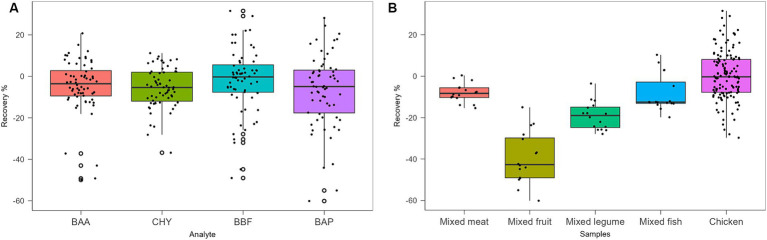
Box Plot of recovery % of analytes **(A)** and samples **(B)**, obtained with Protocol VII.

Protocols from III to VII were tested with chicken baby food samples, performing two replicates for the recovery evaluation at 0.5 μg/kg. Protocols III, IV, V, and VI provided recovery values <10%, as shown in Table 5. Protocol VII provided the best results with recovery values ranging from 70 to 90% and, for this reason, it was chosen as the extraction and cleaning procedure for subsequent validation and monitoring. In particular, concerning other tested procedures, the step at −20°C overnight, together with chosen salts, enhanced the clean-up with better removal of co-extracted fat lipids. Then, protocol VII was also tested on other infant food matrices based on meat, fish, legumes, and mixed fruits. The full dataset obtained from this study was evaluated by using Box Plot, a useful tool of graphic representation used for managing quantitative data. This tool allows the comparison of many distributions in the same graph, highlighting the most significant characteristics such as symmetry, range, variance, and possible outliers. The median value, first and third interquartile, upper and lower limits within Tukey’s limit, and outliers (if any) can be visualized in a single graphical representation. Moreover, the use of median and quartiles in describing the distribution makes the considerations about possible outliers in the dataset more robust. The dataset obtained during the method validation showed similar accuracy parameters for 4 PAHs, but different behavior in different matrices. A more detailed data analysis showed that the mean recovery percentage obtained by analysing baby food samples of mixed meats, legumes, fish products, and chicken were comparable, in the range of 80–100%, with higher variance in chicken samples, while a lower value (~60%) was obtained from homogenized mixed fruits analysis. Regarding method sensitivity, evaluated by elaborating the linear regression, the higher performance was obtained for BAA, followed by CHR, BAP, and BBF. The Box Plot, shown in Figure 2, was successfully applied for optimizing the analytical method for the detection of 4 PAHs in baby food since it allows rapid and effective visualization of all statistical parameters that characterize the procedure.

**Table 5:** Mean values (*N* = 3) of recovery % of smoked salmon samples, spiked at 2 μg/kg, obtained from Protocol III to Protocol VII, using IL8 working standard solution prepared with acetonitrile.

Extraction and clean-up procedure	BAA	BAP	BBF	CHR
*Protocol III*	9.8%	7.3%	9.5%	4.3%
*Protocol IV*	8.8%	7.9%	9.5%	8.9%
*Protocol V*	7.5%	10.1%	10.2%	9.5%
*Protocol VI*	8.3%	7.3%	10.1%	10.2%
*Protocol VII*	70.5%	84.5%	89.2%	71.0%

### 3.2 Method validation

The following validation parameters, namely the linearity of MMC curves, the limit of detection (LOD), the limit of quantification (LOQ), the selectivity, the precision, the recovery percentage, and the ruggedness were determined to assure the efficiency of the proposed GC–MS/MS method and to guarantee the validity of routine analysis. The MMC curve for the 4 PAHs was evaluated from 0.1 to 5 μg/kg, considering the framework of European legislation for residues in baby foods (40). MMC curves were used for the validation method, analyzing each level in triplicate. All validation parameters, obtained using matrix chicken baby food (Table 6**)** resulted in compliance with EU provision No. 836/2011 and 808/2021 (41, 42).

**Table 6:** Validation parameters of BAP, BAA, BBF, and CHR, determined by means of GC–MS/MS analysis of chicken samples, extracted and cleaned-up with Protocol VII.

PAH	*t* s_b_/b < 0.22^a^	Intercept	R^2^^b^	LOD^c^	LOQ^d^	SD ± RSD %^e^	Recovery % ± RSD %^e^	Uncertainty %^f^
BAP	0.056	1.803 × 10^−3^	0.999	0.036	0.110	0.004 ± 5.56	73.08 ± 4.06	10.15
BAA	0.035	3.295 × 10^−3^	0.999	0.023	0.070	0.005 ± 5.43	94.25 ± 5.12	9.98
BBF	0.043	1.457 × 10^−3^	0.999	0.028	0.080	0.005 ± 7.29	76.07 ± 5.54	12.57
CHY	0.030	2.604 × 10^−3^	0.999	0.019	0.060	0.009 ± 7.91	110.70 ± 8.75	13.47

a *t*-Student of the ratios s_b_/b, where s_b_ is the standard deviation of the slope of MMC curve (b).

b *R*^2^is the coefficients of determination of MMC curve.

c Limit of detection (LOD) = 3.3s_a_/b where s_a_ was the standard deviation of the intercept.

d LOQ = 10s_a_/b, both calculated from MMC curve.

e Mean values ± standard deviations (*n* = 6) of blank matrices fortified at 0.1 μg/kg for each analyte.

f Mean values determined of blank matrices fortified at 0.1 μg/kg for each analyte (*n* = 6).

**Figure 3:**
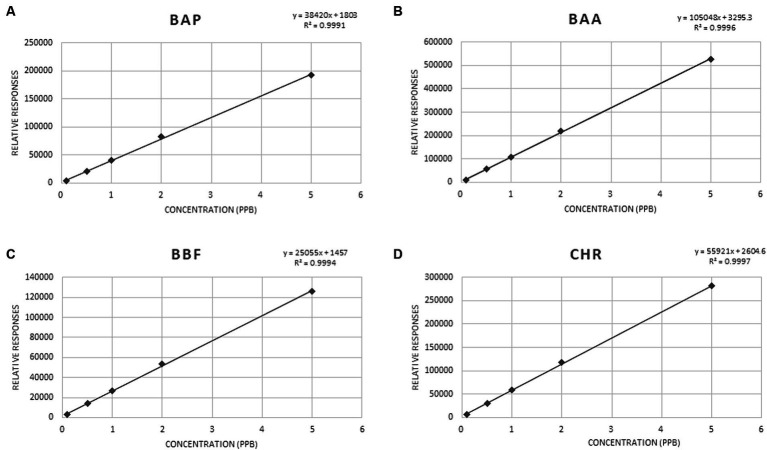
MMC curve for BAP **(A)**, BAA **(B)**, BBF **(C)** and CHR **(D)**.

The coefficients of determination (R^2^ values) were in the range of 0.998–0.999, indicating good calibration linearity in investigated matrices. Mandel test was used to assess whether the data best fitted a linear function. The test was verified with a *p*-value <0.05. The significance of the slope (b), of the regression line obtained from the MMC curve, at *α* = 0.05, was verified with a *t*-test. The *t-Student* was calculated for ratios s_b_/b, where s_b_ was the standard deviation of the slope b, obtained for each of the studied analytes. All the values resulted lower than 0.22. Figure 3 shows MMC curves for each PAHs.

LODs and LOQs were calculated, according to the following equations: LOD = 3.3s_a_/b and LOQ = 10s_a_/b, where s_a_ was the standard deviation of the intercept. The calculated values of LOD and LOQ (Table 6), indicated high sensitivity for the determination of these analytes at trace levels, reducing the risk of false-negative results. Selectivity ensures the correct identification of analytes of interest without interference from other components that could be present in the sample, such as impurities, degradants, matrix components, etc. To verify method selectivity, 20 sample blanks representative of the chosen matrices were analysed. The 20 specificity tests were carried out on the following baby food samples: chicken, beef, lamb, ham, salmon, trout, white bean, chickpea, legumes, pea and courgettes, mixed meat, and mixed fish. The absence of significant interferences in the maximum tolerance range of ±0.1 min for analyte retention times compared to a spiked sample was verified.

**Figure 4:**
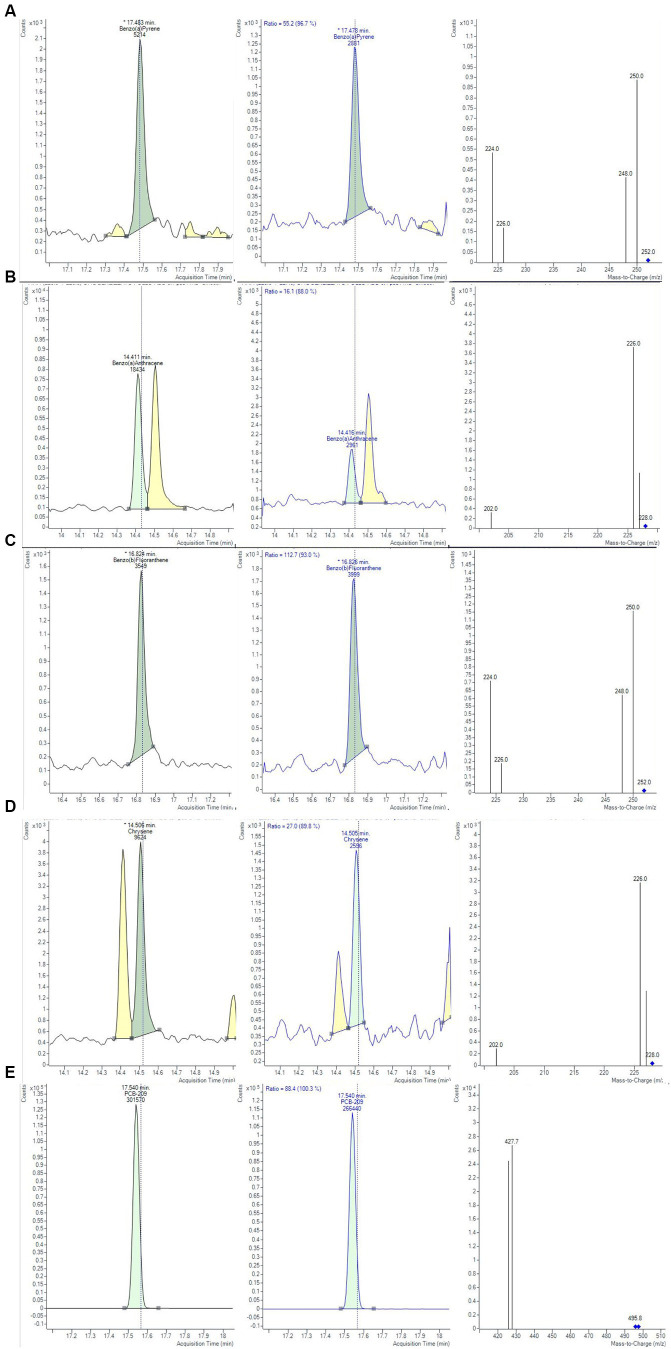
Chromatograms and mass spectra of quantifier and qualifier ions (m/z) used in GC-MS/MS method for **(A)** BAP, **(B)** BAA, **(C)** BBF, **(D)** CHR, **(D)** PCB-209 determination, obtained at 0.1 µg/Kg for the evaluation of method precision.

The trueness and precision of measurements were assessed in accordance with Decision 2021/808/EC (40) through the analysis of spiked samples, prepared starting from blank material by additions of known amounts of the analytes. The precision of a method measures the repeatability, evaluating the degree of agreement among individual test results. The recovery percentage is used to indicate trueness and to express the distance between the mean and the reference value. Precision and trueness were determined by analyzing two sets of blank matrices (six replicates each), spiked at a concentration of 0.1 μg/kg for each analyte. Precision was expressed as deviation standard (SD) and relative deviation standard (RSD), while trueness was expressed as recovery %. The intra-day RSD values were well below the reference values of 20%, derived by the Horwitz equation, under repeatability conditions, demonstrating a good method precision (43). Calculated values of recoveries % and RDS % were in the range of 73–110% and always lower than 8.75%, respectively. Homoscedasticity of data was evaluated using ANOVA (*p* = 0.05), calculating the mean of recoveries %, obtained for the level at 0.1 μg/Kg. The recovery factors resulted from 0.903 to 1.368 for the 4 PAHs. In Figure 4, an example of a chromatographic separation obtained at 0.1 μg/kg was reported, showing the chromatogram of quantifier ions and relative studied transition for all 4 PAHs in chicken baby food samples. For the evaluation of the uncertainty of measurements, the metrological approach was adopted, using the validation data obtained from each step of the analytical procedure. Taking into consideration the uncertainties propagation law, the concentration relative uncertainty was calculated for 4 PAHs, as reported in a previous work (39). A relative expanded measurement uncertainty was calculated using a coverage factor k of 2, corresponding approximately to a 95% confidence level.

The robustness of this analytical method was evaluated by Youden’s test, introducing several changes at once (44). The application of the method to other matrices was considered as changes to be applied. Hence, mixed meat and fish were also spiked at 1.0 μg/Kg of each PAH and then analyzed and compared with chicken samples. The standard deviation of the differences D_i_ (SD_i_) was calculated according to the equation reported by Karageorgou and Samanidou (45). SD_i_ resulted not significantly larger than the standard deviation of the method carried out on validated matrices (chicken samples), confirming that the proposed method is sufficiently robust against the chosen modification.

Moreover, other matrices such as fish and legume baby foods were considered. Further analyses on two sets of blank matrices (four replicates each) were also carried out. Fish and legume blank samples were spiked at a concentration of 0.1 and 1.0 μg/kg for each analyte, respectively. To evaluate the method reproducibility, the absolute difference between independent single test results (C_1_ and C_2_) was considered. The |C_1_-C_2_| values were not found to be higher than the repeatability limit, calculated against the expected 95% probability interval of s_r_ × t√2, where s_r_ is the repeatability standard deviation at 1.0 μg/kg, as shown in Table 7. For fish samples, values of recovery % resulted in the range of 83–105%, while for the legume matrix, they were in the range of 73–85%. Also, these data were compliant with the acceptability criteria of European regulation (41, 42).

**Table 7:** Repeatability and recovery % of BAP, BAA, BBF, and CHR, determined by means of GC–MS/MS analysis of fish and legume baby foods samples, extracted and cleaned-up with Protocol VII.

	Fish baby foods^a^		Legume baby foods^b^	
Analyte	|C_1_-C_2_| < s_r_ × t√2	Recovery %	|C_1_-C_2_| < s_r_ × t√2	Recovery %
BAP	|0.063–0.066| < 0.014	64.5	|0.743–0.756| < 0.400	74.95
BAA	|0.096–0.095| < 0.018	95.5	|0.848–0.859| < 0.233	85.35
BBF	|0.080–0.071| < 0.018	75.5	|0.721–0.742| < 0.334	73.15
CHR	|0.110–0.103| < 0.032	106.5	|0.758–0.822| < 0.214	79.00

a Collected data by performing the analysis on sets of six replicates of blank matrices fortified at a concentration of 0.1 μg/kg for each analyte.

b Collected data by performing the analysis on sets of six replicates of blank matrices fortified at a concentration of 1.0 μg/kg for each analyte.

### 3.3 Evaluation of four PAHs in baby food samples

**Figure 5:**
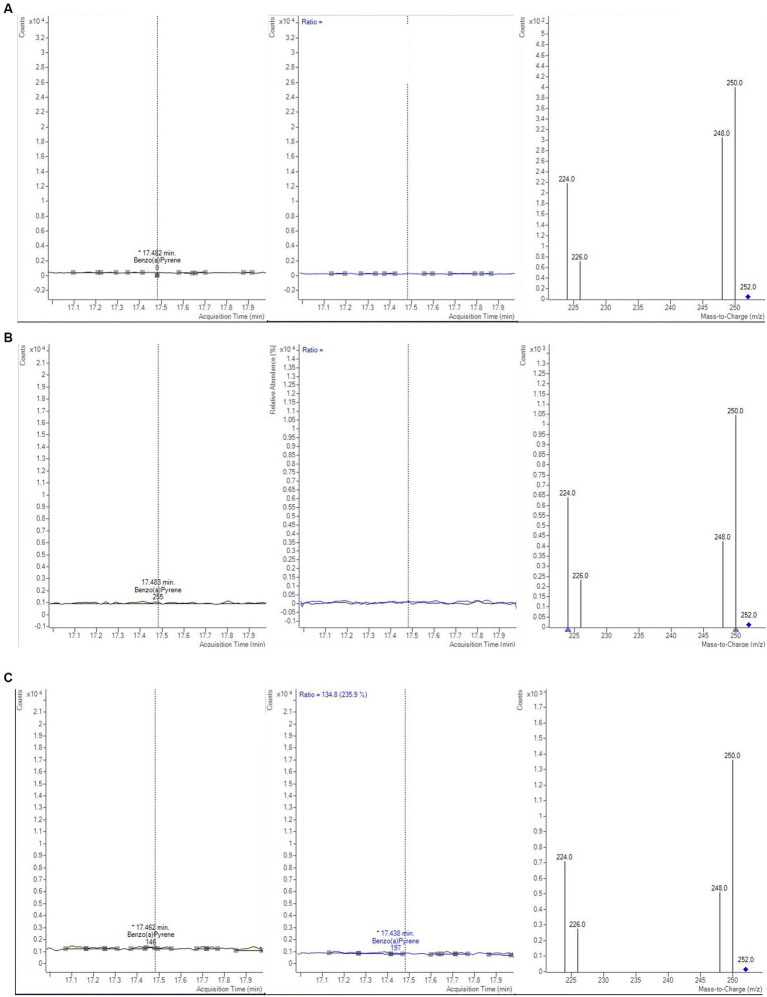
Chromatograms and mass spectra of quantifier and qualifier ions (m/z) used in GC–MS/MS method for BAP determination in fish baby food samples **(A)** not-cooked, **(B)** after microwave cooking and **(C)** after steaming cooking.

The monitoring was performed over 2 years, by analyzing a total of 60 samples of baby food, of common brands present on the Italian market, and of different compositions (meat, fish, legumes, and vegetables). The samples were analyzed in duplicate, verifying that the concentrations of each analyte satisfied the repeatability criteria, i.e., the absolute difference of two values |C1-C2| must not exceed the repeatability limit (s_r_ × t√2). In Table 8 values of s_r_, obtained during the validation step, were calculated at three levels (0.1, 1.0, and 1.5 μg/kg). If |C1-C2| satisfied these criteria, a mean value of concentration was calculated and reported together with its measurement uncertainty, also considering the coverage and recovery factors. The final results were expressed as the sum of 4 PAHs. The results showed that the mean level of total PAHs, for all the investigated samples, was always below the lowest LOQ. Moreover, as reported in the literature, cooking methods could influence the production of PAHs (46). Considering that microwave and steaming cooking are the most used methods to conserve nutrients and quality of baby foods, 40 samples of chicken, salmon, lentils, and mixed legumes and vegetables, were analyzed after these heat treatments. Twenty samples were heated for 5 min in a water bath at 100°C and the other 20 samples were heated for 30 s in a microwave at full power. As in the previous case, PAH contents resulted under the LODs and then they were not detected and quantified (Figure 5). However, in this regard, further studies are needed to evaluate the formation of 4 PAHs also considering other setting parameters (temperature, time, power etc…) but also different cooking treatments.

**Table 8:** Repeatability standard deviation (s_r_) at 0.1, 1.0 and 1.5 μg/kg of BAP, BAA, BBF and CHR, calculated in step of validation method by means of GC–MS/MS analysis of chicken baby foods samples, extracted and cleaned-up with Protocol VII.

s_r_	BAP	BAA	BBF	CHR
0.1 μg/kg	0.004	0.005	0.005	0.008
1.0 μg/kg	0.108	0.064	0.092	0.060
1.5 μg/kg	0.161	0.101	0.053	0.083

## 4 Conclusion

Different extraction and clean-up procedures were evaluated and compared for the analytical determination of four regulated PAHs in different infant foods, aiming to achieve high recovery rates and low detection and quantification limits. The QuEChERS method based on a triple extraction, twice with acetonitrile and once with Bond Elut-EMR lipid dSPE, followed by a subsequent purification with salts NaCl and MgSO_4_ 1:4 and − 20°C overnight, was found to be the best procedure, with recoveries in the range of 70–90% for the target analytes. The proposed method, based on GC–MS/MS analysis, was validated and used to analyze baby food samples (meat, fish, legumes, and vegetables), collected in the Italian market. The monitoring was performed on untreated, steamed, and microwaved baby foods, confirming that the concentration levels of BAP, BAA, BBF, and CHR were lower than LOQs and consequently lower than the EU standard limit (1 μg/kg) in all samples. Moreover, considering the high toxicity and carcinogenicity of BAP, this monitoring has provided reassuring results for children’s common products on the Italian market. In conclusion, the developed GC/MS/MS method provided a highly sensitive and reliable approach for the determination of PAHs in different types of baby foods, even at trace levels. This method can contribute to the assessment of PAH exposure in infants and support regulatory efforts to ensure the safety and quality of infant food products with regular monitoring.

## Data availability statement

The raw data supporting the conclusions of this article will be made available by the authors, without undue reservation.

## Author contributions

MIn: Writing – original draft, Writing – review & editing, Conceptualization, Data curation, Formal analysis, Investigation, Methodology, Software, Validation. RZ: Writing – original draft, Writing – review & editing, Investigation, Visualization. ID: Data curation, Formal analysis, Validation, Writing – original draft. ACh: Data curation, Formal analysis, Writing – original draft, Validation. VN: Conceptualization, Funding acquisition, Investigation, Project administration, Resources, Supervision, Visualization, Writing – original draft. FC: Conceptualization, Data curation, Investigation, Visualization, Writing – original draft. ACa: Formal analysis, Writing – original draft. MQ: Conceptualization, Investigation, Methodology, Visualization, Writing – original draft, Writing – review & editing. DN: Conceptualization, Investigation, Visualization, Writing – original draft. MIa: Conceptualization, Investigation, Project administration, Resources, Supervision, Visualization, Writing – original draft, Writing – review & editing.

## References

[1] Palade LM Negoiță M Adascălului AC Mihai AL. Polycyclic aromatic hydrocarbon occurrence and formation in processed meat, edible oils, and cereal-derived products: a review. Appl Sci. (2023) 13:7877. doi: 10.3390/app13137877

[2] Shoaei F Talebi-Ghane E Amirsadeghi S Mehri F. The investigation of polycyclic aromatic hydrocarbons (PAHs) in milk and its products: a global systematic review, meta-analysis and health risk assessment. Int Dairy J. (2023) 142:105645. doi: 10.1016/j.idairyj.2023.105645

[3] Sampaio GR Guizellini GM da Silva SA de Almeida AP Pinaffi-Langley ACC Rogero MM. Polycyclic aromatic hydrocarbons in foods: biological effects, legislation, occurrence, analytical methods, and strategies to reduce their formation. Int J Mol Sci. (2021) 22:6010. doi: 10.3390/ijms22116010, PMID: 34199457 PMC8199595

[4] Habe H Omori T. Genetics of polycyclic aromatic hydrocarbon metabolism in diverse aerobic bacteria. Biosci Biotechnol Biochem. (2003) 67:225–43. doi: 10.1271/bbb.67.225, PMID: 12728980

[5] Purcaro G Moret S Conte LS. Overview on polycyclic aromatic hydrocarbons: occurrence, legislation and innovative determination in foods. Talanta. (2013) 105:292–305. doi: 10.1016/j.talanta.2012.10.041, PMID: 23598022

[6] Hirano M Tanaka S Asami O. Classification of polycyclic aromatic hydrocarbons based on mutagenicity in lung tissue through DNA microarray. Environ Toxicol. (2013) 28:652–9. doi: 10.1002/tox.20761, PMID: 21887816

[7] Patel AB Shaikh S Jain KR Desai C Madamwar D. Polycyclic aromatic hydrocarbons: sources, toxicity, and remediation approaches. Front Microbiol. (2020) 11:562813. doi: 10.3389/fmicb.2020.562813, PMID: 33224110 PMC7674206

[8] Kang HJ Lee SY Kwon JH. Physico-chemical properties and toxicity of alkylated polycyclic aromatic hydrocarbons. J Hazard Mater. (2016) 312:200–7. doi: 10.1016/j.jhazmat.2016.03.051, PMID: 27037474

[9] Zelinkova Z Wenzl T. The occurrence of 16 EPA PAHs in food – a review. Polycycl Aromat Compd. (2015) 35:248–84. doi: 10.1080/10406638.2014.918550, PMID: 26681897 PMC4673601

[10] European Food Safety Authority. Polycyclic aromatic hydrocarbons in food-scientific opinion of the panel on contaminants in the food chain. EFSA J. (2008) 6:724. doi: 10.2903/j.efsa.2008.724 PMC10193653 37213838

[11] Huertas-Pérez JF Bordajandi LR Sejerøe-Olsen B Emteborg H Baù A Schimmel H. PAHs in baby food: assessment of three different processing techniques for the preparation of reference materials. Anal Bioanal Chem. (2015) 407:3069–81. doi: 10.1007/s00216-015-8490-z, PMID: 25644522 PMC4383830

[12] Darwish WS Chiba H El-Ghareeb WR Elhelaly AE Hui S-P. Determination of polycyclic aromatic hydrocarbon content in heat-treated meat retailed in Egypt: health risk assessment, benzo[a]pyrene induced mutagenicity and oxidative stress in human colon (CaCo-2) cells and protection using rosmarinic and ascorbic acids. Food Chem. (2019) 290:114–24. doi: 10.1016/j.foodchem.2019.03.127, PMID: 31000027

[13] Polachova A Gramblicka T Parizek O Sram RJ Stupak M Hajslova J. Estimation of human exposure to polycyclic aromatic hydrocarbons (PAHs) based on the dietary and outdoor atmospheric monitoring in the Czech Republic. Environ Res. (2020) 182:108977. doi: 10.1016/j.envres.2019.108977, PMID: 31821985

[14] Duan X Shen G Yang H Tian J Wei F Gong J. Dietary intake polycyclic aromatic hydrocarbons (PAHs) and associated Cancer risk in a cohort of Chinese urban adults: inter- and intra-individual variability. Chemosphere. (2016) 144:2469–75. doi: 10.1016/j.chemosphere.2015.11.019, PMID: 26619312 PMC4695288

[15] Hokkanen M Mikkelä A Pasonen P Tuominen P Uusitalo L Erkkola M. Children’s dietary exposure to polycyclic aromatic hydrocarbons in Finland. Polycycl Aromat Compd. (2022) 42:4651–65. doi: 10.1080/10406638.2021.1903951

[16] Huang X Deng X Li W Liu S Chen Y Yang B. Internal exposure levels of polycyclic aromatic hydrocarbons in children and adolescents: a systematic review and meta-analysis. Environ Health Prev Med. (2019) 24:50. doi: 10.1186/s12199-019-0805-9, PMID: 31351468 PMC6661086

[17] Moazzen M Shariatifar N Arabameri M Hosseini H Ahmadloo M. Measurement of polycyclic aromatic hydrocarbons in baby food samples in Tehran, Iran with magnetic-solid-phase-extraction and gas-chromatography/mass-spectrometry method: a health risk assessment. Front Nutr. (2022) 9:833158. doi: 10.3389/fnut.2022.833158, PMID: 35252309 PMC8891379

[18] Mielech A Puścion-Jakubik A Socha K. Assessment of the risk of contamination of food for infants and toddlers. Nutrients. (2021) 13:2358. doi: 10.3390/nu13072358, PMID: 34371868 PMC8308760

[19] Drwal E Rak A Gregoraszczuk EL. Review: polycyclic aromatic hydrocarbons (PAHs)—action on placental function and health risks in future life of newborns. Toxicology. (2019) 411:133–42. doi: 10.1016/j.tox.2018.10.003, PMID: 30321648

[20] Bansal V Kim K-H. Review of PAH contamination in food products and their health hazards. Environ Int. (2015) 84:26–38. doi: 10.1016/j.envint.2015.06.016, PMID: 26203892

[21] European Commission. Commission regulation (EU) 2023/915 of 25 April 2023 on maximum levels for certain contaminants in food and repealing regulation (EC) no 1881/2006. OJEU. (2023) L 119) 66:103–57.

[22] Agus BAP Rajentran K Selamat J Lestari SD Umar NB Hussain N. Determination of 16 EPA PAHs in food using gas and liquid chromatography. J Food Compos Anal. (2023) 116:105038. doi: 10.1016/j.jfca.2022.105038

[23] Zianni R Mentana A Campaniello M Chiappinelli A Tomaiuolo M Chiaravalle AE. An investigation using a validated method based on HS-SPME-GC-MS detection for the determination of 2-dodecylcyclobutanone and 2-tetradecylcyclobutanone in X-ray irradiated dairy products. LWT. (2022) 153:112466. doi: 10.1016/j.lwt.2021.112466

[24] Anastassiades M Lehotay SJ Štajnbaher D Schenck FJ. Fast and easy multiresidue method employing acetonitrile extraction/partitioning and “dispersive solid-phase extraction” for the determination of pesticide residues in produce. J AOAC Int. (2003) 86:412–31. doi: 10.1093/jaoac/86.2.412, PMID: 12723926

[25] He Z Wang L Peng Y Luo M Wang W Liu X. Multiresidue analysis of over 200 pesticides in cereals using a QuEChERS and gas chromatography–tandem mass spectrometry-based method. Food Chem. (2015) 169:372–80. doi: 10.1016/j.foodchem.2014.07.102, PMID: 25236240

[26] Santana-Mayor A Rodríguez-Ramos R Herrera-Herrera AV Socas-Rodríguez B Rodríguez-Delgado MA. Updated overview of QuEChERS applications in food, environmental and biological analysis (2020–2023). Trends Anal Chem. (2023) 169:117375. doi: 10.1016/j.trac.2023.117375

[27] Perestrelo R Silva P Porto-Figueira P Pereira JAM Silva C Medina S. QuEChERS - fundamentals, relevant improvements, applications and future trends. Anal Chim Acta. (2019) 1070:1–28. doi: 10.1016/j.aca.2019.02.036, PMID: 31103162

[28] Surma M Sadowska-Rociek A Cieślik E. The application of d-SPE in the QuEChERS method for the determination of PAHs in food of animal origin with GC–MS detection. Eur Food Res Technol. (2014) 238:1029–36. doi: 10.1007/s00217-014-2181-4

[29] Anastassiades M Scherbaum E Taşdelen B Štajnbaher D. Recent developments in QuEChERS methodology for pesticide multiresidue analysis. Pesticide Chem. (2007) 46:439–58. doi: 10.1002/9783527611249.ch46

[30] Lehotay SJ. Collaborators: determination of pesticide residues in foods by acetonitrile extraction and partitioning with magnesium sulfate: collaborative study. J AOAC Int. (2007) 90:485–520. doi: 10.1093/jaoac/90.2.485, PMID: 17474521

[31] Sun Y Wu S. Analysis of PAHs in oily systems using modified QuEChERS with EMR-lipid clean-up followed by GC-QqQ-MS. Food Control. (2020) 109:106950. doi: 10.1016/j.foodcont.2019.106950

[32] Chen B-H Inbaraj BS Hsu K-C. Recent advances in the analysis of polycyclic aromatic hydrocarbons in food and water. J Food Drug Anal. (2022) 30:494–522. doi: 10.38212/2224-6614.3429, PMID: 36753366 PMC9910297

[33] Sadowska-Rociek A Surma M Cieślik E. Comparison of different modifications on QuEChERS sample preparation method for PAHs determination in black, green, red and white tea. Environ Sci Pollut Res Int. (2014) 21:1326–38. doi: 10.1007/s11356-013-2022-1, PMID: 23900956 PMC3880489

[34] ITeh Stand (n.d.) EN 16619:2015 - food analysis - determination of benzo[a]pyrene, benz[a]anthracene, chrysene and benzo[b]fluoranthene in foodstuffs by gas chromatography mass spectrometry (GC-MS). Available at: https://standards.iteh.ai/catalog/standards/cen/ca6d5bc8-3a90-4857-a250-7cfcb9096c4b/en-16619-2015 (Accessed January 19, 2024).

[35] Wenzl T Simon R Anklam E Kleiner J. Analytical methods for polycyclic aromatic hydrocarbons (PAHs) in food and the environment needed for new food legislation in the European Union. Trends Anal Chem. (2006) 25:716–25. doi: 10.1016/j.trac.2006.05.010

[36] Prata R López-Ruiz R Nascimento LES Petrarca MH Godoy HT Frenich AG. Method validation for GC-measurable pesticides and PAHs in baby foods using QuEChERS-based extraction procedure. J Food Compos Anal. (2024) 129:106062. doi: 10.1016/j.jfca.2024.106062

[37] European Commission 2013/609/E commission regulation (EU) 2013/609 regulation (EU) of 12 June 2013 on food intended for infants and young children, food for special medical purposes, and total diet replacement for weight control and repealing council directive 92/52/EEC, commission directives 96/8/EC, 1999/21/EC, 2006/125/EC and 2006/141/EC, directive 2009/39/EC of the European Parliament and of the council and commission regulations (EC) no 41/2009 and (EC) no 953/2009 (text with EEA relevance) (2023) Available at: http://data.europa.eu/eli/reg/2013/609/2023-03-21

[38] Nardelli V D’Amico V Della Rovere I Casamassima F Marchesiello WMV Nardiello D. Box Behnken design-based optimized extraction of non-dioxin-like PCBs for GC-ECD and GC-MS analyses in milk samples. Emerg Contam. (2020) 6:303–11. doi: 10.1016/j.emcon.2020.08.002

[39] Nardelli V D’Amico V Ingegno M Della Rovere I Iammarino M Casamassima F. Pesticides contamination of cereals and legumes: monitoring of samples marketed in Italy as a contribution to risk assessment. Appl Sci. (2021) 11:7283. doi: 10.3390/app11167283

[40] European Commission. 2002/657/EC Commission decision of 12 august 2002 implementing council directive 96/23/EC concerning the performance of analytical methods and the interpretation of results (notified under document number C(2002) 3044). OJEU. (2002) 45:8–36.

[41] European Commission. 2011/836/EC Commission regulation (EU) no. 836/2011 amending regulation (EC) no. 333/2007 laying down the methods of sampling and analysis for the official control of the levels of lead, cadmium, mercury, inorganic tin, 3-MCPD and benzo(a)pyrene in foodstuffs. OJEU. (2011) 4:9–116.

[42] European Commission. 2021/808/EC Commission implementing regulation (EU) 2021/808 of 22 march 2021 on the performance of analytical methods for residues of pharmacologically active substances used in food-producing animals and on the interpretation of results as well as on the methods to be used for sampling and repealing decisions 2002/657/EC and 98/179/EC (text with EEA relevance). OJEU. (2021) 64:84–109.

[43] Thompson M. Recent trends in inter-laboratory precision at ppb and sub-ppb concentrations in relation to fitness for purpose criteria in proficiency testing. Analyst. (2000) 125:385–6. doi: 10.1039/B000282H

[44] César IDC Pianetti GA. Robustness evaluation of the chromatographic method for the quantitation of lumefantrine using Youden’s test. Braz J Pharm Sci. (2009) 45:235–40. doi: 10.1590/S1984-82502009000200007

[45] Karageorgou E Samanidou V. Youden test application in robustness assays during method validation. J Chromatogr A. (2014) 1353:131–9. doi: 10.1016/j.chroma.2014.01.050, PMID: 24508395

[46] Siddique R Zahoor AF Ahmad H Zahid FM Karrar E. Impact of different cooking methods on polycyclic aromatic hydrocarbons in rabbit meat. Food Sci Nutr. (2021) 9:3219–27. doi: 10.1002/fsn3.2284, PMID: 34136186 PMC8194747

